# Molecular Tools for the Detection and the Identification of Hymenoptera Parasitoids in Tortricid Fruit Pests

**DOI:** 10.3390/ijms18102031

**Published:** 2017-09-22

**Authors:** Pierre Franck, Mariline Maalouly-Matar, Jérôme Olivares

**Affiliations:** INRA, UR1115, Plantes et Systèmes de culture Horticoles, F-84914 Avignon CEDEX 9, France; marilinemaalouly@gmail.com (M.M.-M.); jerome.olivares@inra.fr (J.O.)

**Keywords:** *Cydia*, *Grapholita*, parasitoid wasps, molecular identification, parasitism level, parasitoid interaction, *Ascogaster*, *Perilampus*, *Pristomerus*, *Trichomma*

## Abstract

Biological control requires specific tools for the accurate detection and identification of natural enemies in order to estimate variations in their abundance and their impact according to changes in environmental conditions or agricultural practices. Here, we developed two molecular methods of detection based on PCR-RFLP with universal primers and on PCR with specific primers to identify commonly occurring larval parasitoids of the tortricid fruit pests and to estimate parasitism in the codling moth. Both methods were designed based on DNA sequences of the *COI* mitochondrial gene for a range of parasitoids that emerged from *Cydia pomonella* and *Grapholita* *molesta* caterpillars (102 parasitoids; nine species) and a range of potential tortricid hosts (40 moths; five species) damaging fruits. The PCR-RFLP method (digestion by AluI of a 482 bp *COI* fragment) was very powerful to identify parasitoid adults and their hosts, but failed to detect parasitoid larvae within eggs or within young *C. pomonella* caterpillars. The PCR method based on specific primers amplified *COI* fragments of different lengths (131 to 463 bp) for *Ascogaster quadridentata* (Braconidae); *Pristomerus* *vulnerator* (Ichneumonidae); *Trichomma enecator* (Ichneumonidae); and Perilampus tristis (Perilampidae), and demonstrated a higher level of sensibility than the PCR-RFLP method. Molecular estimations of parasitism levels in a natural *C. pomonella* population with the specific primers did not differ from traditional estimations based on caterpillar rearing (about 60% parasitism in a non-treated apple orchard). These PCR-based techniques provide information about within-host parasitoid assemblage in the codling moth and preliminary results on the larval parasitism of major tortricid fruit pests.

## 1. Introduction

With the shift towards a reduced reliance on external inputs in agriculture, identifying management options that enhance pest control services has become a critical issue [1]. The successful implementation of pest management programs requires a better understanding of the ecology behind the provision of ecosystem services [2] and methods to detect and identify the biodiversity linked with these services [3]. Such methods are crucial when selecting biocontrol candidates [4] and when evaluating the efficiencies of biocontrol releases [5] or the impact of changes in agricultural practices [6]. They are also very helpful to quantify the trophic interactions within a community of natural enemies and the impact of the food web structure on the control of herbivore pests [7].

The rapid expansion and the public availability of referenced DNA barcode sequences [8] has favoured the development of numerous PCR-based techniques to analyse the predation and parasitism of arthropod pests [9,10]. PCR-amplified fragments can be subjected to RFLP, and sequenced or directly sized for species identification [3]. For example, these PCR-based techniques have been used to identify the presence of pests in contaminated food [11,12], to reveal prey in the gut or the faeces of predators [13], to determine the host from which a parasitoid adult emerged [14], or to detect parasite larvae inside their host [15]. These PCR-based techniques allow for an accurate estimation of parasitism levels in pest collected in the field, including the identification of multi- and hyper-parasitism [16]. They are at least as efficient as the traditional method of detection, which requires insect rearing during long periods or careful host dissection [17].

The codling moth, *Cydia pomonella* (L.) (Lepidoptera: Tortricidae), is one of the major insect pests in temperate regions, damaging several cultivated fruit trees, notably apple orchards [18]. The codling moth female lays about 100 single eggs, mainly on the upper side of the host-tree leaves. A free neonate larva hatches from each egg and penetrates into a fruit. At the end of its development, the caterpillar leaves the fruit, and, depending on the temperature and photoperiod conditions, either pupates to give an adult or enters into diapause. Depending on the latitude, the codling moths complete one to four generations per year. Insecticide treatments remain the major means used to avoid fruit damage and to maintain codling moth populations at a low level. As a consequence of these treatments, *C. pomonella* has developed resistances to many chemical and biological insecticides [19,20,21]. This demonstrates the need to develop alternative methods and to diversify management programs to control this pest.

Hymenoptera parasitoids, because of their relatively high specificity to a host species, have long been recognized as important agents in the biological control of insect pests in agriculture [10,22]. Globally, the parasitism of the tortricid pests remains low (usually less than 5% in commercial crops), but several hymenopteran parasitoids have been described to emerge from various codling moth instars (for a review, see Mills 2005 [23]). In Europe, the three most abundant parasitoids that have emerged from collections of diapausing codling moth caterpillars in apple orchards are *Ascogaster quadridentata* (Braconidae), *Pristomerus vulnerator* (Ichneumonidae), and *Perilampus tristis* (Perilampidae) [24,25,26,27]. *Bassus rufipes* (Braconidae) and *Trichomma enecator* (Ichneumonidae) have also been frequently recorded, notably from caterpillars collected on walnut trees [23]. The pupal or prepupal parasitoids *Dibrachys cavus* (Pteromalidae), *Hyssopus palidus* (Eulophidae), *Mastrus rufipes*, and *Liotryphon caudatus* (Ichneumonidae) have been infrequently observed [23,28].

The biology and the ecology of these parasitoids remains largely unknown. The braconid wasp, *A. quadridentata*, is an ovo-larval endoparasitoid specialized on the tortricid moths [24]. The parasitized codling moth caterpillar is significantly smaller than the non-parasitized one [29]. It is killed at the fourth instar, and the parasitoid larva finalizes its development as an ectoparasite in a smooth cocoon within the codling moth cocoon [30]. The ichnomonid wasp, *P. vulnerator*, is an endoparasitoid attacking a young caterpillar just after it enters into the fruit. The parasitoid larva remains latent until the caterpillar leaves the fruit for pupation [31]. It then weaves a hard elongated cocoon to finalize its development as an ectoparasite [25]. The species is reported as stenophagous, and develops on several Lepidoptera hosts [25,27]. Cases of super-parasitism or multi-parasitism were reported for some *Pristomerus* species [32]. The chalcid wasp, *P. tristis*, has been described as a hyperparasitoid [33]. It parasitizes several tortricid primary parasitoids, but preferentially the braconids [25,34,35]. The *Perilampus* planidium stays inactive within the caterpillar until the primary parasitoid has reached the pupal stage. It then penetrates its host body to finalize its development [30].

There are finally quite a few parasitoid species involved in the codling moth parasitism, and their detection within their host could be made easier using molecular techniques. Here, we report the development of two different PCR-based techniques that can provide rapid, accurate, and cost-effective detection and identification of the most common parasitoid species associated with codling moth caterpillars. We tested both techniques for their ability to detect the parasitoid species at different stages of their development within the codling moth and their reliability for parasitism evaluation in natural *C. pomonella* populations.

## 2. Results

### 2.1. DNA Barcoding

**Figure 1:**
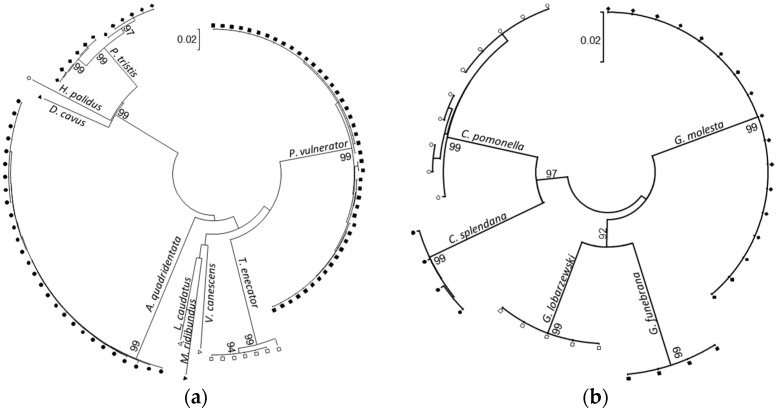
Neighbor-Joining trees (Saitou and Nei 1987 [36]) of (**a**) the codling moth Hymenoptera parasitoids (97 specimens, nine different genera) and (**b**) of their potential tortricid hosts (40 specimens, five different species). The evolutionary distances between coding sequences (651 positions) were computed using the Kimura two-parameter method (Kimura 1980 [37]). The confidence probabilities that the interior branch length is greater than zero (Dopazo 1994 [38]) were estimated using bootstrap tests (2000 replicates) and were represented next to the branches for a probability above 90%.

The 102 sequenced parasitoids were identified as *P. vulnerator* (Ichneumonidae: Cremastinae; 45 specimens), *A. quadridentata* (Braconidae: Cheloninae; 28 specimens), *T. enecator* (Ichneumonidae: Anomaloninae; 7 specimens), *P. tristis* (Perilampidae; 12 specimens), *B. rufipes* (Braconidae: Agathidinae; 5 specimens), *H. palidus* (Eulophidae), *D. cavus* (Pteromalidae), *Mastrus ridibundus* (Ichneumonidae: Cryptinae), *L. caudatus* (Ichneumonidae: Pimplinae), and *Venturia canescens* (Ichneumonidae: Campopleginae), one specimen for each of these five last species (Supplementary Table S1). The five individuals recognized as *Bassus rufipes* had an identical non-coding *COI*-like sequence (KP402060–KP402064), which likely corresponds to a nuclear mitochondrial pseudogene. The 97 remaining specimens had *COI*-coding mitochondrial sequences (KP072518–KP072654). All the DNA sequences matched with *COI* sequences recorded in BOLD with similarities above 90% (Supplementary Table S1). At the species level, BOLD confirmed the identifications of all the *A. quadridentata*, *D. cavus*, *L. caudatus*, and *V. canescens* specimens (DNA similarities above 99.5% with identified sequences). The *T. enecator* DNA sequences matched with non-identified Ichneumonidae (similarities above 99.2%). The *M. ridibundus* DNA sequence matched with the DNA sequences of several *Isadelphus* (100% similarities), a related genus within the Cryptinae. The net nucleotide divergences within each taxon ranged between 0.2% in *A. quadridentata* and 1.1% in *P. tristis*. Two mitotype groups, differing by less than 2%, were detected both in *T. enecator* and in *P. tristis* (Figure 1a). Nucleotide divergences between taxa ranged between 11% (divergence between *P. tristis* and *D. cavus*) and 26% (divergence between *A. quadridentata* and *M. ridibundus*), and exceeded 17% in average between taxa (Figure 1a). Comparatively, the nucleotide divergences among the 40 tortricid specimens (Figure 1b, Supplementary Table S1) ranged from 9% to 12% (within and among the *Cydia* and *Grapholita* genus, respectively). The net nucleotide divergence among the *C. pomonella* specimens (0.4%) was twice higher than the divergence among specimens within each of the other tortricid species. These parasitoid and moth barcodes were used to design two simple molecular methods for estimating parasitism rate in the codling moth and for identifying their parasitoids.

### 2.2. Parasitoid and Moth Identification Using PCR-RFLP

The PCR-RFLP method was designed in order to identify each parasitoid species and their tortricid hosts (Table 1). The diagnosis was based on the sizes of the restriction fragments at both extremities of the amplified fragment using fluorescent-marked PCR primers. The *Cat0* and *Nancy* primers and the AluI restriction enzyme were chosen to optimize the diagnosis among species. No intra-specific polymorphism was detected. Different RFLP patterns were observed between the five tortricid species and between eight out of nine parasitoid species. The PCR-RFLP test was not able to differentiate *P. tristis* from *D. cavus*. The PCR-RFLP pattern observed with *B. rufipes* was specific, but corresponded to the restriction of a non-coding *COI*-like sequence. Different RFLP patterns were observed between the parasitoid species and the tortricid species. The differences were mainly due to a difference in the number of restriction fragments (one to five fragments in the Hymenoptera species; three to six in the Lepidoptera species).

### 2.3. Parasitoid Identification Using Specific PCR Primers

**Figure 2:**
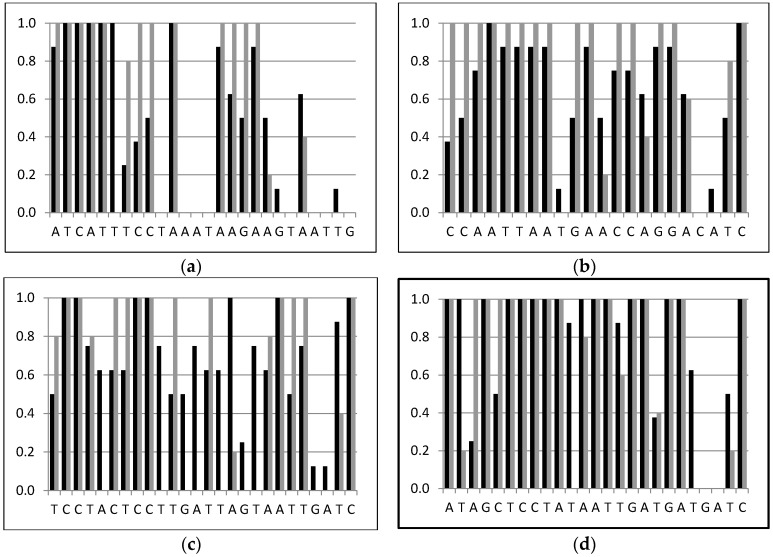
Similarity comparison between the specific primers *Asco* (**a**); *Peri* (**b**); *Pristo* (**c**); and *Tricho* (**d**) and the non-specific parasitoid (black) or the tortricid host (grey) sequences.

Specific reverse primers in the *COI* gene region were designed to be used with fluorescent-marked *LCO-1490* in the four most abundant parasitoid species that emerged from codling moth caterpillars: *A. quadridentata*, *P. vulnerator*, *T. enecator*, and *P. tristis*. Up to five different primers were tested in combination with *LCO-1490* for each parasitoid and host species. The *Asco*, *Pristo*, *Peri*, and *Tricho* primers (Table 2) were finally selected for their high specificity (Figure 2). They respectively amplified a *COI* fragment of 131, 347, 124 and 463 bp in *A. quadridentata*, *P. vulnerator*, *P. tristis*, and *T. enecator*. Each primer amplified the expected fragment on the parasitoid species for which it was designed, but not on other parasitoid and tortricid species (respectively nine and five species tested). Note, however, that the *Tricho* primer amplified a 463 bp fragment on *M. ridibundus* but the signal did not reach 5% of the signal intensity observed in *T. enecator*.

### 2.4. Early Detection A. quadridentata in Parasitized Codling Moth

Parasitism by *A. quadridentata* was estimated on ten independent infestation experiments on 35 codling moth eggs. Each parasitoid female had exploited all the eggs available 40 min after it was introduced into the petri dish arena. In this artificial condition, codling moth mortality rates were significantly higher during the first week of development (mean = 0.079, SD = 0.013) than later (mean = 0.032, SD = 0.001, Chi^2^ = 22.5, df = 1, *p* = 5 × 10^−4^). However, the mortality of immature instars did not differ between parasitized and non-parasitized caterpillars (respectively 0.227 and 0.289, Chi^2^ = 1.14, df = 1, *p* = 0.307). Parasitism levels were estimated with both the PCR-RFLP method and the *Asco* specific primer on the different codling moth instars (Table 3). Estimates of parasitism levels in immature instar with the *Asco* primer did not significantly differ from 85%, the average proportion of parasitoid adult that emerged from codling moth caterpillars (parasitism level: mean = 0.867, SD = 0.046, Chi^2^ = 0.0651, df = 1, *p* = 0.83). Estimates of the parasitism level in immature codling moth instar were significantly lower with the PCR-RFLP method than with the *Asco* specific primer (Table 3). The PCR-RFLP method is likely to have failed in amplifying the parasitoid DNA at an early parasitism stage in its host.

### 2.5. Detection of Parasitism in Naturally Occurring Codling Moth Population

Parasitism was estimated in a non-treated apple orchard on 184 mature caterpillars (47% with weight lower than 30 mg). Of these caterpillars, 123 were monitored to determine parasitism based on adult emergences (Table 4). The molecular detection and identification of parasitoids was performed within their hosts for the remaining 61 caterpillars. The PCR-RFLP tests only detected a few caterpillars that were parasitized by *A. quadridentata* (corresponding to only 54% of the caterpillars detected positive with the *Asco* primer) and no other parasitoid species, suggesting that this method is not as sensitive as the specific method. Consequently, the molecular identification of the parasitoids within the codling moth caterpillars was only based on the *Asco*, *Pristo*, *Peri*, and *Tricho* specific primers. The same three parasitoid species—*A. quadridentata*, *P. vulnerator*, and *P. tristis*—were both observed to emerge (Table 4) and were molecularly recorded (Table 5). No difference was observed between parasitism levels detected with the traditional rearing method and the specific PCR-based method (respectively 0.595 and 0.598, Chi^2^ = 0.0082, df = 1, *p* = 0.99). Similarly, there was no difference between the two methods in the distribution of the parasitoid species (Chi^2^ = 2.67, df = 3, *p* = 0.43). Thirty-eight percent (38%) of the parasitized caterpillars were parasitized by a single parasitoid species, 95% of which were *A. quadridentata*. Hyperparasitism by *P. tristis* accounted for 55% of the parasitized caterpillars, 84% of which were also PCR-positive for *A. quadridentata*. Parasitism by *P. vulnerator* accounted for 8% of the parasitized caterpillars, 75% of which were also PCR-positive for *A. quadridentata*. Finally, 5% of the parasitized caterpillars were PCR-positive for all the three species simultaneously (Table 5). Mortality before emergence did not differ between the small and large caterpillars (respectively 0.316 and 0.318, Chi^2^ = 0.0429, df = 1, *p* = 0.84, Table 4). However, parasitism levels were four times higher in the small caterpillars than in the large ones (all the small caterpillars were detected as parasitized). The parasitoid species composition also differed between both categories of caterpillar sizes (Chi^2^ = 37.2, df = 10, *p* = 5 × 10^−4^, Table 5). Ninety-eight percent (98%) of the small caterpillars were parasitized by *A. quadridentata*, whereas only 71% of the large caterpillars were positive for this species.

## 3. Discussion

In this study, molecular tools were developed to identify hymenopteran parasitoids in some important tortricid pests attacking fruits. The method, based on the digestion of a fragment of the *COI* gene, proved very useful to identify parasitoid and hyperparasitoid adults and their Lepidoptera or Hymenoptera hosts. The use of individual primer sets to amplify uniquely sized fragments of the four most abundant *C. pomonella* parasitoids—*A. quadridentata*, *P. vulnerator*, *P. tristis*, and *T. enecator*—provided an accurate estimation of parasitism within immature Lepidoptera hosts (egg or caterpillar); the molecular assessment of parasitism levels did not differ from estimates based on the monitoring of the emergences of parasitoid and moth adults, but was able to reveal the occurrence of multi-parasitism within the codling moth caterpillars and identify the species involved in this interaction, which is not possible with traditional rearing.

### 3.1. Molecular Identification of the Hymenoptera Parasitoids

#### 3.1.1. Species Delimitation Based on Barcoding Sequences

The ten hymenopteran parasitoid species observed to emerge from *C. pomonella* and *Grapholita molesta* caterpillars collected in apple orchards from Western and Central Europe were all clearly differentiated according to their DNA barcodes. DNA variations observed within these morphologically identified species were lower than 2%, the accepted threshold to delimit species according to *COI* barcode sequences [41,42]. Note, however, that two groups of DNA sequences differing by more than 1% were clearly observed in both the *P. tristis* and *T. enecator* taxa. These clusters differentiated specimens collected in the same apple orchard. Species that cannot be differentiated based on their mitochondrial DNA sequences have been identified in numerous taxa [43,44,45]. For example, in the *Cotesia melitaearum* parasitoid complex (Hymenoptera: Braconidae), host-associated species were identified only based on microsatellite markers [46]. Genetic differentiation according to host specialization should be further investigated to check the presence of cryptic species within these parasitoid taxa, notably in *P. tristis* that parasitizes both *A. quadridentata* and *P. vulnerator* primary parasitoids within the tortricid caterpillars. Similarly, the only collected specimen identified as *M. ridibundus* had a DNA barcode that matched 100% with specimens referenced as other Cryptinae species from the *Isadelphus* genus. Although we cannot exclude some errors, either in our morphological identification or those reported in BOLD, this result mainly suggests that barcoding only based on *COI* sequences would be not efficient enough to differentiate some closed parasitoid taxa. The sequencing of other mitochondrial and nuclear genes would be useful to confirm species identification and delimitation among the tortricid parasitoids. Finally, the molecular identification of *B. rufipes* remains limited in the absence of a barcoding reference for this taxon to compare with those of the other parasitoid species. Re-sequencing using new sets of primer sequences would be helpful to obtain the *COI* barcode of *B. rufipes*.

#### 3.1.2. Parasitoid Identification Based on Simple Diagnostic Molecular Tests

*COI* sequences provided information to identify the most important functional groups of parasitoids attacking *C. pomonella* and *G. molesta* caterpillars. The PCR-RFLP method developed for routine identification was efficient enough to differentiate eight out of the ten morphologically observed parasitoid species. The two non-differentiated species based on this PCR-RFLP test, *P. tristis* and *D. cavus*, belong to two different families of chalcid wasps that cannot be mistaken in terms of taxonomy and biology (Perilampidae and Pteromalidae, respectively). The pteromalid wasp is a polyembryonic parasitoid that attacks mature caterpillars within their cocoon. Conversely, *P. tristis* is a solitary hyperparasitoid that parasitizes young caterpillars [23]. However, the *Cat0* and *Nancy* primers used with the PCR-RFLP method failed to amplify early *A. quadridentata* stages within its host. This is likely the result of PCR competition when amplifying parasitoid and tortricid DNAs with the same primers, which is exacerbated for a low DNA amount. The primers designed to specifically amplify *A. quadridentata*, *P. vulnerator*, *P. tristis*, and *T. enecator*, the most abundant parasitoid species observed within the tortricid caterpillars, permitted a more complete diagnosis. None of the primers amplified a species other than that for which they were designed. These specific primers appear to be more sensitive than the PCR-RFLP method, and the *Asco* primer demonstrated the ability to detect *A. quadridentata* in very early stages of parasitism within the codling moth eggs. Although this set of specific primers provides a limited diagnosis of the parasitoid species attacking *C. pomonella* caterpillars in apple orchards, the four parasitoids detected represented more than 95% of the larval parasitism in the natural codling moth populations in the present study. These parasitoids are also the species most observed to attack *C. pomonella* caterpillars in Europe [23,25].

### 3.2. Molecular Method for Lepidoptera Host Identification

The identification of tortricid larvae within damaged fruits remains of major concern for quarantine decisions both in America and in Europe, notably the ability to differentiate between indigenous and exotic species [11]. Several simple methods for the identification of tortricid species based on *COI* sequencing were developed based on the use of specific PCR primers [11] or on PCR-RFLP approaches [11,47,48]. Such molecular methods are indeed useful to differentiate between tortricid species for which morphological larval identification remains problematic due to a lack of diagnostic characters [49]. The PCR-RFLP diagnosis presented here was based on the restriction digestion by a single enzyme (AluI). It is an efficient alternative to the methods previously published, which are based on the use of several restriction enzymes to identify the tortricid pests. The method provides the added benefit of identifying key parasitoids and their host range after the rearing of parasitized caterpillars, which often cannot be identified morphologically because of size modifications associated with parasitism [29].

### 3.3. Molecular Characterization of the Parasitoid Communities

The expansion of DNA barcode references largely contributes to the development of the construction of parasitoid food webs and the analysis of trophic interactions [50,51]. Species identifications directly based on barcode sequences have been used to detect potential links between parasitoids and their hosts [14,52,53,54], and the proportion of papers based on these metabarcoding approaches are likely to increase quickly with the development of Next Generation Sequencing [55]. Metabarcoding approaches are very powerful to identify an assemblage of parasitoid species within their hosts without any a priori knowledge of potential interaction links between species. However, metabarcoding approaches still require important bioinformatics development to properly quantify trophic interactions, and are not as cheap, fast, and easy to use as the traditional barcoding method [56,57]. The simple molecular methods developed here were based on an a priori knowledge of the parasitoid community. They are limited to the identification of a predefined set of parasitoids, but can be very useful to understand the interactions among species and differences between agronomic or environmental conditions in a well-defined biological system [58,59]. The molecular methods proposed here to assess codling moth parasitism provided similar results to those obtained with the traditional methods of caterpillar rearing, suggesting that the results would be comparable. Furthermore, these molecular methods highlighted the complex relationships among the parasitoid species inside their codling moth caterpillar hosts. First, they revealed that parasitism by *T. vulnerator* mainly required a pre-infestation by *A. quadridentata.* Second, they confirmed that *P. tristis* parasitized both the *A. quadridentata* and *T. vulnerator* primary parasitoid inside the codling moth caterpillars [25], revealing a few cases of parasitized caterpillars by the three parasitoid species. Third, the molecular methods also confirmed that the size reduction of the codling moth caterpillars was mainly due to parasitism by *A. quadridentata* [29], which accounted for about 92% of the total *C. pomonella* parasitism in the non-treated apple orchard analysed. This result suggests that the proportion of small caterpillars could be a valuable way to primarily estimate codling moth parasitism level.

### 3.4. Molecular Estimation of Parasitism Levels

Parasitism levels are usually estimated from a collection of insect larvae either based on the ratio of parasitoid adults that emerged, which requires insect rearing and emergence monitoring, or based on the proportion of larvae detected as parasitized, which can be assessed directly within the larvae using molecular markers. We estimated parasitism levels using both methods. Estimates based on specific PCR detection of the four most abundant parasitoid species inside the codling moth caterpillars did not significantly differ from estimates based on caterpillars rearing for adult emergences [17,26]. This result contrasts with previous comparative studies, which usually detected higher parasitism levels with molecular methods than with the traditional ones [15,60,61]. We suggest two possible explanations for the differences in parasitism detection between methods and insect instars. First, age-dependent variation in parasitism levels could result from early mortality of the parasitized larvae because of the stings and venoms used by the parasitoid wasps to immobilize their hosts [17]. Second, detecting the presence of parasitoid DNA within its host does not warrant functional parasitism, since numerous insects developed immune defence base on the encapsulation of the parasitoid eggs within their body [62,63]. The main codling moth parasitoid, *A. quadridentata*, directly lays its egg within the egg of its host, thereby potentially avoiding the encapsulation process. Laboratory parasitization of codling moth eggs by *A. quadridentata* did not detect mortality differences between the parasitized and non-parasitized caterpillars, which is also in agreement with the absence of observed differences in the estimates of parasitism levels between methods. Interestingly, these observations suggest that we could compare the various estimates of parasitism in the codling moth populations independently of the methodology used. Consistently with previous reports [26,64], the parasitism of *C. pomonella* caterpillars was significantly higher in non-treated apple orchards (up to 60%) than in commercial ones (usually less than 5%).

### 3.5. Detection of Parasitism and Potential for Codling Moth Biological Control

The present study confirms that the parasitism of the *C. pomonella* caterpillar in the apple orchard is mainly due to the braconid, *A. quadridentata*. The other observed parasitoid species were either rare or acted as superparasites that attack *A. quadridentata*-parasitized codling moth caterpillars (e.g., *P. vulnerator* and *P. tristis*). This superparasitism does not enhance pest mortality, and it likely limits the growth of *A. quadridentata* populations and codling moth biocontrol [23,65]. The molecular tools we developed allow for the identification of parasitoid species that are not too much vulnerable to hyperparasitoid attack to be used for augmentative release and exploring agronomic and environmental conditions that may limit hyperparasitism [66]. The molecular methods developed here should be very useful to extend parasitism comparison among the tortricids, both within crops and in semi-natural habitats.

## 4. Material and Methods

### 4.1. Biological Material Used as References

The biological material used to develop the molecular identification techniques encompassed 102 parasitoid adults that emerged from tortricid caterpillars (92 and 10 caterpillars were recognized as *C. pomonella* and *G. molesta*, respectively) and 40 non-parasitized tortricids moths considered as potential host species for these parasitoids (see Supplementary Table S1). This material was collected in various orchards in western and central Europe between 2002 and 2013. One half of the parasitoid adults was part of a previously published study on the codling moth parasitism in the Basse–Durance Valley, France [26]. The other half was from caterpillars collected in organic or non-treated orchards in France (Rhône-Alpes, Occitanie, and Normandie regions), the Czeck Republic (Hradec-Králové), and Germany (Bade-Wurtemberg). The tortricid moths emerged from caterpillars collected on apple (*C. pomonella*, *G. molesta*, *G. lobarzewkii*), plum (*G. funebrana*), and chestnut (*C. splendana*) trees from the same French regions as the collected parasitoids. The emerging parasitoids and moths were conserved in 95% ethanol until identification and DNA extraction. The parasitoids were morphologically identified using different keys [24,67] and taxonomic descriptions [68,69,70,71]. The tortricid moths were identified based on male genitalia morphology [72].

### 4.2. DNA Barcode Sequences

DNA barcode sequences of the 102 hymenoptera parasitoids and of the 40 tortricid moths were obtained using standard protocols for DNA isolation, PCR amplification, DNA sequencing, sequence editing, and sequence alignment [73]. DNA was isolated from single individuals in 200 µL 10% Chelex 100 (Bio-Rad, Hercules, CA, USA) resin solution including 50 µg proteinase K (Eurobio, Les Ulis, France) according to Walsh et al. [74]. PCR amplifications were performed with the *LCO1490* and *HCO2198* universal primers [40] (Table 1) using the GoTaq Flexi DNA polymerase kit (Promega, Madison, WI, USA) in 25 µL PCR reaction volume containing 1X Flexi^®^ buffer, 1 unit of *Taq* polymerase, 200 µM of each dNTP, 1.5 mM MgCl_2_, 0.1 mg/mL BSA, 0.4 µM of each primer, and 2 µL of DNA template. PCR samples were subjected to an initial denaturation of 2 min at 94 °C, then 30 cycles including 30 s at 95 °C, 45 s of annealing at 48 °C, and 45 s of elongation at 72 °C. Bidirectional DNA sequencing was conducted with the *LCO1490* and *HCO2198* primers using the Big Dye Terminator v3.1 kit (Applied Biosystems, Foster City, CA, USA) and an ABI3730XL sequencer on purified PCR products by treatment with exonuclease I and phosphatase at Genoscope (Evry, France). *LCO1490* and *HCO2198* reads on electropherograms were compared to correct, assemble, and finally edit the DNA barcode sequences with BioEdit v7.2.1 [75]. Species identification of each DNA barcode sequence were performed on the Barcode of Life Data System (BOLD identification System for *COI*, release 5.25) [76]. The DNA barcode sequences were analysed with MEGA, v6.0.6 [77]. Parasitoid and moth multiple DNA sequences were aligned separately using ClustalW, v2.0 [78], and translated in amino acid sequences to detect non-coding sequences. Neighbor-joining trees [36] were computed with the Kimura two-parameter distance [37] between pairs of DNA barcode sequences to illustrate intra- and interspecific nucleotide divergences in the parasitoid and moth taxa.

### 4.3. Parasitoid and Moth Identification Using PCR-RFLP

Restriction maps of each DNA barcode sequences were edited with BioEdit to select diagnostic restriction enzymes and restriction sites able to differentiate the parasitoid and moth species. The AluI enzyme was chosen to develop a PCR-RFLP identification technique because it had few restriction sites (AG^CT) in the 3′ end of the barcode region, which were at different positions for the set of parasitoid and moth species to identity (one to five restriction sites per species). PCR amplification of this diagnostic barcode region (476–482 bp) was performed with the primers *Cat0* and *Nancy* [39] labeled with HEX and ATTO565 dyes, respectively (Table 1). The primer *Cat0* (Table 1) was redesigned from the *Ron* primer, 5′-GGATCACCTGATATAGCATTCCC-3′ [39]. The *Cat0* sequence was established to match the barcode sequences of both the tortricid moths and their parasitoids, and to avoid the presence of an AluI restriction site within the primer. Different dyes were attached to the PCR primers to allow species diagnosis only based on the RFLP lengths of the two external RFLP fragments. PCR amplifications were performed in 12 µL reaction volumes containing 10 mM Tris-HCl, pH 9, 50 mM KCl, 200 µM of each dNTP, 0.4 µM of each primer, 1.5 mM MgCl_2_, one unit Taq DNA polymerase (Promega), 0.1 mg/mL BSA with 2 µL of DNA template. After an initial denaturing step of 2 min at 94 °C, 30 cycles were performed consisting of 30 s at 95 °C, 45 s of annealing at 48 °C, and 45 s of elongation at 72 °C. PCR products were subsequently cut overnight at 37 °C in 20 µL reaction volumes with one unit of the AluI (NEB, Ipswich, MA, USA) restriction enzyme. Digested fragments were visualized after electrophoresis on an ABI3730 DNA sequencer. This method was developed to co-identify the parasitoids and their hosts in a single PCR run.

### 4.4. Parasitoid Identification Using Specific PCR Primers

Specific reverse PCR primers were designed in the barcode sequences of each of the most abundant parasitoids on *C. pomonella*: *A. quadridentata*, *P. vulnerator*, *P. tristis*, and *T. enecator*, to amplify relatively short amplicons with *LCO1490.* Before their use, primer specificity had been verified in silico using Primer-BLAST [79] analyzing site-by-site similarities with the barcodes of other parasitoid and moth species. Then, independent PCR tests were conducted with each designed primer on moth and parasitoid specimens from the reference set to estimate their reliabilities to amplify the parasitoid species for which they were designed. For each specific primer, PCRs were performed in the same condition as for the PCR-RFLP method, but using 54 °C as annealing temperature and with a PCR buffer including 20 mM ammonium sulfate. The *LCO1490* primer was labeled with FAM dye to visualize the amplified fragments on an ABI3730 DNA sequencer. A PCR test was assumed as positive for any amplification at the expected size that was higher than 5% of the signal intensity in the parasitoid controls. This method was developed for a more accurate estimation of the parasitism in immature codling moth.

### 4.5. Early Detection of A. quadridentata Parasitism in Immature Codling Moth

Codling moth eggs were parasitized in laboratory condition to estimate parasitism detection at various codling moth instars with both the PCR-RFLP and the primer specific approaches. We infested 10 sets of 35 codling moths eggs with *A. quadridentata*. For each set, a virgin female wasp was transferred into a Petri dish arena containing the host eggs for approximately 2 h at 25 °C. Then, we removed the parasitoid female and a sample of five eggs was immediately collected. We subsequently collected five neonates, five young, and five aged caterpillars, after respectively one, two, and three weeks of development. The collected samples were killed and individually conserved at −20 °C. Finally, we monitored the emergence of the remaining materials. All the collected caterpillars were placed in individual vials at 25 °C with a 16 h day length with a nutritive soybean instant diet (Stonefly Heliothis diet, Ward’s, Rochester, NY, USA) prepared in aqueous solution with 0.2% acetic acid. Similarly, we used five sets of 35 non-infested eggs as control. These sets were monitored in the same conditions as the infested eggs to estimate mortality of each codling moth instar in the absence of parasitoid. DNA of each codling moth instar—infested and non-infested—was extracted using the DNeasy Tissue Kit (Qiagen, Hilden, Germany) following the manufacturer’s instructions, and were screened to detect parasitism by *A. quadridentata*. For each *C. pomonella* instar, Pearson’s chi-square tests were performed with R (*p*-value simulated based on 2000 replicates) [80] to respectively compare (i) mortality between parasitized and control samples and (ii) parasitism detection between the PCR-RFLP and the primer specific methods.

### 4.6. Parasitism Detection in Naturally Occurring Codling Moth Populations

We analyzed a naturally infested codling moth population in order to compare parasitism levels using both molecular and traditional approaches. Mature codling moth caterpillars were collected in November 2009 with band traps in a non-treated apple orchard (Gotheron, France, 44°58′21.13″ N, 4°55′38.70″ E). The collected caterpillars were divided into two sets according to their weight (above and below 30 mg). About one third of the caterpillars of each set were directly killed to estimate parasitism only based on the molecular methods. The remaining larvae were stored in individual vials in an outdoor insectarium during the winter and left to naturally emerge [17]. The emerging parasitoids were morphologically identified [24]. Their cocoons and those of their host were also carefully inspected to identify primary and secondary hosts [25]. Each sample (caterpillars, parasitoids, and their empty-host cocoons) were conserved in 90% ethanol before DNA extraction. DNA of each sample was extracted separately using 200 µL of 10% Chelex 100 (Bio-Rad) resin solution [74]. The PCR-RFLP method was used to determine the parasitoid adults and their tortricid hosts. PCRs were also performed with each of the four parasitoid specific primers to identify the parasitized cocoons and caterpillars and subsequently determine the parasitoid assemblage within each individual host. Parasitism levels were estimated as the ratio of emerging parasitoids on the total of emerging moths and parasitoids (traditional method) or as the ratio of caterpillars detected positive for at least one PCR test (molecular method). Pearson’s chi-square tests were performed to respectively compare: (i) estimates of parasitism rates and (ii) distributions of the parasitoid species both between the small and large caterpillar sets and between the traditional and the molecular approaches.

## Figures and Tables

**Table 1:** DNA lengths (in bp) for each parasitoid and host species resulting from the PCR-RFLP in the *COI* mitochondrial gene. Restriction fragment lengths were ranged according to their position along the *COI* sequence. The PCR-RFLP diagnosis of the parasitoids and their tortricid hosts was based on the lengths of the two external fragments, which were labeled with different dyes attached to the forward and reverse PCR primers. Undetected restriction fragments are provided in brackets.

Species	Trophic Level	Total Length	Restriction Fragment Lengths
*Cydia pomonella*	Host	482	170-(45)-(78)-(15)-174
*Cydia splendana*	Host	482	170-(45)-(78)-(48)-141
*Grapholita molesta*	Host	482	156-(59)-(78)-(15)-(33)-141
*Grapholita funebrana*	Host	482	191-(24)-(78)-189
*Grapholita lobarzewskii*	Host	482	191-(24)-(78)-(15)-174
*Ascogaster quadridentata*	Parasitoid	482	347-135
*Pristomerus vulnerator*	Parasitoid	482	191-(135)-156
*Trichomma enecator*	Parasitoid	482	215-(186)-81
*Perilampus tristis*	Hyperparasitoid	476	335-(6)-135
*Mastrus ridibundus*	Parasitoid	482	215-(111)-(15)-(6)-135
*Liotryphon caudatus*	Parasitoid	482	170-(21)-(135)-156
*Dibrachys cavus*	Parasitoid	476	335-(6)-135
*Hyssopus palidus*	Parasitoid	476	335-141
*Venturia canescens*	Parasitoid	482	482-482
*Bassus rufipes*	Parasitoid	480	170-(45)-(126)-139 ^a^

^a^ Restriction length of a non-coding *COI*-like pseudogene.

**Table 2:** Description of the PCR primers used to amplify different parts of the *COI* gene for the identification of the codling moth parasitoids. Primers were named according to the nomenclature of Simon et al. (1994) [39] in *Drosophila melanogaster* and their alias (in brackets). DNA barcodes were obtained with the universal primers *LCO1490* and *HCO2198* [40] used both for PCR and sequencing. The PCR-RFLP technique was conducted with the primers *Cat0* (redesigned in this study from *Ron*) and *Nancy* [39]. Specific PCR amplifications of the parasitoids were conducted with *LCO1490* as forward primer and different specific reverse primers: *Asco*, *Pristo*, *Tricho*, and *Peri* (designed in this study).

Primers	Sens	5′–3′ Sequences	Identification Techniques
C1-J-1464 (*LCO1490*)	Forward	GGTCAACAAATCATAAAGATATTGG	Barcode
C1-N-2172 (*HCO2198*)	Reverse	TAAACTTCAGGGTGACCAAAAAATCA	Barcode
C1-J-1757 (*Cat0*)	Forward	CCTGATATAGCATTTCCTCG	PCR-RFLP
C1-N-2191 (*Nancy*)	Reverse	CCCGGTAAAATTAAAATATAAACTTC	PCR-RFLP
AQ-C1-N-1595 (*Asco*)	Reverse	ATCATTTCCTAAATAAGAAGTAATTG	*Ascogaster*
PV-C1-N-1811 (*Pristo*)	Reverse	TCCTACTCCTTGATTAGTAATTGATC	*Pristomerus*
TE-C1-N-1927 (*Tricho*)	Reverse	ATAGCTCCTATAATTGATGATGATC	*Trichomma*
PT-C1-N-1588 (*Peri*)	Reverse	CCAATTAATGAACCAGGACATC	*Perilampus*

**Table 3:** Average estimates of parasitism rates by *A. quadridentata* in various codling moth instars (age in weeks) with two alternative PCR methods (diagnosis based on RFLP or with the *Asco* specific primer). Pearson’s chi-square tests were performed to compare estimates of the parasitism rate between both methods.

Host Instar	Age	*N*	RFLP	Specific	Chi^2^	*p*-Value
egg	0	48	0.00	0.90	77.89	5 × 10^−4^
neonate	1	44	0.09	0.89	55.71	5 × 10^−4^
young larvae	2	50	0.07	0.80	52.60	5 × 10^−4^
old larvae	3	41	0.76	0.88	2.03	0.255
adult	>4	67	0.85	0.85	0.00	0.999

**Table 4:** Distribution of the parasitoids that emerged from codling moth larvae collected in an apple orchard (traditional method). *N* and *n* respectively indicate (i) the number of codling moth larvae and (ii) the number of emerging moths and parasitoids that were analyzed for two classes of caterpillar sizes. Estimates of the parasitism levels correspond to the ratio of the number of emerging parasitoid on *n*.

Host Larva Sizes	*N*	*n*	*Ascogaster quadridentata*	*Pristomerus vulnerator*	*Perilampus tristis* on		Parasitism Level
					*Ascogaster*	*Pristomerus*	
>30 mg	66	45	1	1	3	6	24%
<30 mg	57	39	16	3 ^a^	20	0	100%

^a^ One *P. vulnerator* emerged from a larvae also containing an *A. quadridentata* cocoon.

**Table 5:** DNA detection of parasitoid assemblage within 184 codling moth caterpillars collected in an apple orchard (123 out of 184 caterpillars were left to emerge). Estimates of the parasitism levels were based on the ratio of positive PCR for any parasitoids on the total number of collected codling moth caterpillars (97 were large, >30 mg, and 86 were small caterpillars, <30 mg, respectively).

Parasitoid Combination	>30 mg	<30 mg
*A. quadridentata*	3	37
*P. vulnerator*	1	1
*P. vulnerator + A. quadridentata*	4	3
*P. tristis + A. quadridentata*	6	42
*P. tristis + P. vulnerator*	6	1
*P. tristis + P. vulnerator + A. quadridentata*	4	2
Parasitism level	25%	100%

## Supplementary Materials

Supplementary materials can be found at www.mdpi.com/1422-0067/18/10/2031/s1.
